# Interactive digital interventions to promote self-management in adults with asthma: systematic review and meta-analysis

**DOI:** 10.1186/s12890-016-0248-7

**Published:** 2016-05-23

**Authors:** Gary McLean, Elizabeth Murray, Rebecca Band, Keith R. Moffat, Peter Hanlon, Anne Bruton, Mike Thomas, Lucy Yardley, Frances S. Mair

**Affiliations:** Institute of Health and Wellbeing, University of Glasgow, Glasgow, G12 9LX UK; Research Department of Primary Care and Population Health, University College London, Rowland Hill Street, London, UK; Academic Unit of Psychology, Faculty of Social and Human Sciences, University of Southampton, Southampton, UK; Faculty of Health Sciences, University of Southampton, Southampton, UK; Primary Care & Population Sciences, Aldermoor Health Centre, Aldermoor Close, Southampton, UK; NIHR Southampton Respiratory Biomedical Research Unit, Southampton, UK; NIHR Wessex CLAHRC, Southampton, UK

## Abstract

**Background:**

To identify, summarise and synthesise the evidence for using interactive digital interventions to support patient self-management of asthma, and determine their impact.

**Methods:**

Systematic review with meta-analysis. We searched MEDLINE, EMBASE, CINAHL, PsycINFO, ERIC, Cochrane Library, DoPHER, TROPHI, Social Science Citation Index and Science Citation Index. The selection criteria requirement was studies of adults (16 years and over) with asthma, interventions that were interactive digital interventions and the comparator was usual care. Outcomes were change in clinical outcomes, cost effectiveness and patient-reported measures of wellbeing or quality of life. Only Randomised Controlled Trials published in peer-reviewed journals in English were eligible.

Potential studies were screened and study characteristics and outcomes were extracted from eligible papers independently by two researchers. Where data allowed, meta-analysis was performed using a random effects model.

**Results:**

Eight papers describing 5 trials with 593 participants were included, but only three studies were eligible for inclusion for meta-analysis. Of these, two aimed to improve asthma control and the third aimed to reduce the total dose of oral prednisolone without worsening control. Analyses with data from all three studies showed no significant differences and extremely high heterogeneity for both Asthma Quality of Life (AQLQ) (Standardised Mean Difference (SMD) 0.05; 95 % Confidence Interval (CI) 0.32 to −0.22: I2 96.8) and asthma control (SMD 0.21; 95 % CI −0.05 to .42; I2 = 87.4). The removal of the third study reduced heterogeneity and indicated significant improvement for both AQLQ (SMD 0.45; 95 % CI 0.13 to 0.77: I2 = 0.34) and asthma control (SMD 0.54; 95 % CI 0.22 to 0.86: I2 = 0.11). No evidence of harm was identified.

**Conclusion:**

Digital self-management interventions for adults with asthma show promise, with some evidence of small beneficial effects on asthma control. Overall, the evidence base remains weak due to the lack of large, robust trials.

**Electronic supplementary material:**

The online version of this article (doi:10.1186/s12890-016-0248-7) contains supplementary material, which is available to authorized users.

## Background

Asthma is a common condition affecting an estimated 300 million people worldwide and is increasing in prevalence in many countries [1]. Sub-optimal control of asthma is common, with patient adherence to regular preventer medication such as inhaled corticosteroids (ICS) reported to be as low as 30 % [1], potentially leading to increased symptoms, increased risk of asthma attacks and reduced quality of life [2, 3]. Patient education and proactive self-management have been shown to improve clinical outcomes in people with asthma [4, 5]. Guided self-management for asthma is aimed at improving knowledge of the condition and increasing the ability of an individual to manage variations in their asthma [6]. This offers the potential for an improved quality of life, as well as reductions in hospitalisations, emergency room visits, asthma attacks, unscheduled visits to the doctor, and days off work in those with asthma [7]. It is estimated that only a quarter of asthma patients have a self-management plan, despite evidence for the benefits of having one [8]. In most healthcare systems asthma is predominantly managed in primary care. However, primary care support for self-management in asthma can be sub-optimal [9].

One potential method for improving self-management is through the use of interactive digital interventions (IDIs), which offer the possibility of enabling patients to self-manage long-term conditions such as asthma and hence improve outcomes [10–12]. IDIs are packages that can combine health information with decision support to help inform behaviour change in patients, and are typically delivered through the internet or via smart phones. They offer the potential to improve the lives of people with asthma through automating and personalising routine aspects of education, monitoring and support, whilst at the same time giving patients convenient 24 h access to detailed, personalised feedback [13, 14]. There is evidence that well-designed IDIs can change patient health-related behaviour, improve patient knowledge and increase confidence for self-management of health problems [10, 11, 15].

There is little work assessing the impact of IDIs on asthma outcomes. Previous reviews of internet-based interventions have generally focused on telemedicine, where support may not necessarily be interactive or tailored to the user, finding that these interventions improved medication adherence [16] but did not improve asthma symptom scores [17]. Studies that have focused solely on self-management IDIs in those with asthma have included both children and adults and not restricted comparisons to usual care [18, 19]. Results of these reviews suggested that IDIs improved markers of self-care and knowledge, but evidence of improvement in clinical outcomes such as lung function were less clear [18, 19]. The successful implementation of IDIs into primary care will depend, at least in part, on their benefits and cost-effectiveness being clearly demonstrated to primary care clinicians [20, 21]. Therefore we undertook this systematic review to identify, summarise and synthesise the evidence for using IDIs to support patient self-management of asthma, and determine their impact on clinical outcomes, control and knowledge of asthma, quality of life, medication adherence, health service utilisation and cost-effectiveness.

## Methods

### Design

Systematic review and meta-analysis.

A registered protocol (PROSPERO 2014: CRD42014013455) guided the conduct of this review [22], which is reported in adherence to the Preferred Reporting Items for Systematic Reviews and Meta-analyses (PRISMA) Statement [23].

### Eligibility criteria

Inclusion criteria were based on the PICOS (Population, Intervention, Comparator, Outcomes, Study type) acronym (http://library.med.nyu.edu/library/instruction/handouts/pdf/picohandout.pdf). The population was adults with asthma. We defined adults as people aged 16 years and over. Where studies included participants below 16 years of age, the study was only included if we were able to extract the data on those aged 16 or over. The intervention was an interactive digital intervention (as defined below); the comparator was usual care; outcomes were objectively measured change in clinical outcomes and / or patient-reported outcomes of wellbeing or quality of life; and the study type was Randomised Controlled Trials (RCTs) as they present the strongest level of evidence. Finally, we only considered studies published in peer-reviewed journals in English as evidence suggests that limiting studies in this way does not introduce significant bias [24] but does save considerable resource.

For the purpose of this review the term IDIs will include any intervention accessed through a computer (work or home), or smartphone or other hand held device and include web based programmes, desktop computer programmes or apps that provide self-management information and can be used on or offline. The intervention must function without the need for directive input from a health professional. They must also be ‘interactive’, which we define as requiring contributions from programme users (e.g. entering personal data, making choices) which alter pathways within programmes to produce tailored material and feedback that is personally relevant to users.

### Information sources and search strategy

Searches were undertaken by the York Health Economic Consortium, a professional systematic review company (http://www.yhec.co.uk/). The strategies were informed by the intervention search terms used in a previous systematic review conducted by the team on digital asthma self-management interventions [18]. The search strategy combined 3 concepts and a study type filter for RCTs:

1. Asthma
2. Digital interventions
3. Self-management/behaviour change/patient experience
4. RCTs

The following databases were searched: MEDLINE, EMBASE, CINAHL, PsycINFO, ERIC, Cochrane Library (including CDSR, DARE, Central, NHS EED and HTA databases), DoPHER and TROPHI (both produced by the EPPI Centre), Social Science Citation Index and Science Citation Index. These databases were searched using a combination of subject headings where available (such as MeSH) and words in the title and abstracts. The search strategy for MEDLINE is available in Additional file 1 and was adapted for use in the other databases.

The search was complemented by contacting experts in the topic under review and by carrying out citation searches for included studies [25].

### Study selection

Following de-duplication, all abstracts identified from the search were downloaded into the Distiller software programme (https://distillercer.com/products/distillersr-systematic-review-software/). Abstracts and full papers were screened by two reviewers working independently against the inclusion criteria. Inter-reviewer disagreements were resolved by discussing whether the paper met the inclusion / exclusion criteria. If consensus between the reviewers was not possible, the decision was referred to the steering group.

### Data extraction

We used online data collection forms using Distiller SR software. Two independent researchers extracted data on study details (country of origin, inclusion/exclusion criteria, number of participants), participant details (mean age, % male, ethnicity, socio-economic status, smoking status and comorbidities), intervention details (description, theoretical basis, setting, duration, intensity and format) and outcomes. Outcomes were classified into clinical outcomes (asthma control, symptoms (e.g. diary card scores), lung function); asthma-related quality of life; behavioural outcomes (e.g. medication adherence): cognitive outcomes (knowledge of condition, satisfaction with care); affective outcomes (change in depression or anxiety); and economic outcomes (use of health service resources, costs of intervention).

### Assessment of methodological quality

Risk of bias was assessed in each of the included studies by the two researchers working independently, using the Cochrane collaboration tool for assessing bias for guidance [26]. Methods of allocation concealment, randomisation procedure, dropout rate and whether there was evidence of selective outcome reporting were assessed.

### Analysis of interventions

Meta-analysis was based on guidelines from the Cochrane Handbook for Systematic Reviews of Interventions [27]. Potential publication bias was assessed by using a funnel plot and Egger’s test [28]. Where the quantity and quality of data permitted, we undertook meta-analysis, using standardised mean differences and a random effects model (DerSimonian–Laird method) [27]. Where several papers reported the same study, we took the one with the longest duration of follow-up. Where standard deviation of the change was not reported we estimated the standard deviation using confidence intervals or p-values [27]. Heterogeneity statistics were assessed by the Q statistic and I^2^ statistics [29]. For the Q statistic, *p* < 0.10 was considered to indicate statistically significant heterogeneity. The I^2^ statistic indicates the percentage of the observed between-study variability due to differences in study populations, interventions or methods, rather than chance, with the following suggested ranges: no heterogeneity, I^2^ = 0–25 %; moderate heterogeneity, I^2^ = 25–50 %; large heterogeneity, I^2^ = 50–75 %; and extreme heterogeneity, I^2^ = 75–100 % [27].

## Results

**Fig. 1:**
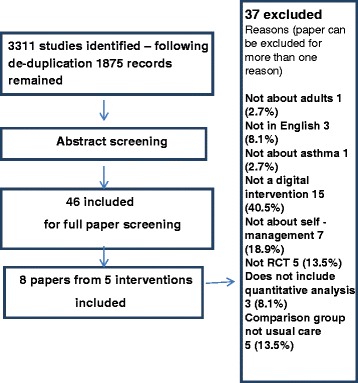
PRISMA Flowchart depicting the study selection procedure

Our search identified 1875 unique citations of which 46 required full paper review. Eight papers reporting five studies with 593 participants met our criteria and were included (see Fig. 1).

### Description of Included studies

Of the eight included papers, four papers reported from the SMASHING study [30–33] For this study, one paper was selected as the primary data source for the meta-analysis, based on length of follow-up) [30]. Where necessary, data from this publication were supplemented with data from accompanying papers (e.g. for a more detailed description of the intervention).

The five included studies had a total of 593 participants (range 50 – 200) (Table 1). In addition to the SMASHING study one further intervention was undertaken in the Netherlands [34]. The remaining studies were conducted in the USA [35], Taiwan [36] and Denmark [37]. The studies varied considerably in the nature and delivery of the intervention, the study population, and the outcome measures used.

**Table 1:** Characteristics of included papers

Author (Year)	Definition of Asthma	Population Numbers (I = Intervention, C = Control)	Mean	Ethnicity (I = Intervention, C = Control	N (%)	Outcomes assessed	Main results
			Age (years)		Males		
Location			(I = Intervention, C = Control		(I = Intervention, C = Control		
van Gaalen [30] (2013) Netherlands	physician-diagnosed prescription of inhaled corticosteroids ≥3 months in the previous year	I =47, C = 60	I = 37.0 C = 36.	N/A	I = 12 (26), C = 19 (32)	AQLQ, ACQ, Symptom-free days, FEV, daily inhaled corticosteroids(DCID	At 30 months after baseline, a sustained and significant difference in terms of asthma-related quality of life of 0.29 (95 % CI 0.01-0.57) and asthma control of −0.33 (95 % CI −0.61 to −0.05) was found in favor of the Intervention group. No sig differences were found for FEV2 or daily inhaled corticosteroids
Van der Meer [31] (2009) Netherlands	Physician-diagnosed prescription of inhaled corticosteroids ≥3 months in the previous year	I = 99 C = 101	I = 37.0 C = 36.0	N/A	I = 29(29) C = 32 (32)	Asthma knowledge, Inhaler technique, Self-reported medication adherence, Physician visits, Telephone contacts, medication changes, AQLQ, ACQ, Symptom-free days, FEV, DCID	Asthma-related quality of life showed a greater increase in the intervention group(adjusted between-group difference, 0.38 [95 % CI, 0.20 to 0.56]). . Asthma control improved more in the I group than in the UC group (adjusted difference, _0.47 [CI, _0.64 to _0.30]).
Van der Meer [32] (2010) Netherlands	physician-diagnosed prescription of inhaled corticosteroids ≥3 months in the previous year	I = 99 C = 101 (subgroups ) well controlled asthma (I = 37, C = 38), partly controlled asthma (I = 38, C = 33), Uncontrolled asthma (I = 26, C = 28)	well controlled asthma (I = 35.8, C = 36.7), partly controlled asthma (I = 35.5, C = 36.3), Uncontrolled asthma (I = 36.9, C = 36.0)	N/a	well controlled asthma (I = 11(29.7 %), C = 12(31.6 %)), partly controlled asthma (I = 8 (21.1 %) C = 10 (30.3)), Uncontrolled asthma I = 13(50.0 %) C = 7 (25.0)	Self-reported medication adherence, , medication changes,ACQ, daily inhaled corticosteroids(DCID)	Improvements in ACQ score after 12 months were −0.14 (*p* = 0.23), −0.52 (*p* < 0.001) and −0.82 (*p* < 0.001) in the intervention group compared to usual care for patients with well, partly and uncontrolled asthma at baseline, respectively. Daily inhaled corticosteroid dose significantly increased in the Internet group compared to usual care in the first 3 months in patients with uncontrolled asthma (+278 μg, *p* = 0.001
Van der Meer [33] (2011) Netherlands	physician-diagnosed prescription of inhaled corticosteroids ≥3 months in the previous year	I = 99 C = 101	I = 37.0 C = 36.0	N/A	I = 29(29) C = 32 (32)	quality adjusted life years (QALYs), costs for health care use and absenteeism	QALYs did not statistically significantly differ between the Intervention group and usual care. Costs of the Internet-based intervention were $254 (95 % CI, $243 to $265) during the period of 1 year. From a societal perspective, the cost difference was $641 (95 % CI, $21957 to $3240). From a health care perspective, the cost difference was $37 (95 % CI, $2874 to $950).
Hashimoto [34] (2011) Netherlands	diagnosis of severe refractory asthma according to the major and minor criteria recommended by the American Thoracic Society	I = 51 C = 38	I = 48.5 C = 52.4	n/a	I = 23 (45) C = 18 (47)	AQLQ, ACC, Slope FEV1, Exacerbations, Days of hospitalisation per patient, ICS, Sparing of oral corticosteroids, prednisone dose	Median cumulative sparing of prednisone was 205 (25-75th percentile 221 to 777) mg in the internet strategy group compared with 0 (497 to 282) mg in the conventional treatment group (*p* = 0.02).
Bender [35] (2012) USA	Physician diagnosed asthma for which they were prescribed daily inhaled corticosteroid treatment	I = 25, C = 25	I = 39.6 C = 43.5	I = 56 % white, 24 % Hispanic, 20 % African American, 0 % Asian. C = 60 % white, 12 % Hispanic, 20 % African American, 0 % Asian.	I = 10 (40 %) C = 8(32 %)	Medication Adherence, Belief in Medications Questionnaire, AQLQ, ACT	No differences emerged for the AQLQ or ACT**.** Adherence was 32 % higher in intervention group and increased score in belief in medication was found for intervention group
Liu [36] (2011) Taiwan	moderate-to-severe persistent asthma based on criteria for asthma as defined by the American Thoracic Society on the basis of clinical symptoms and physical examination.	I = 43 C = 46	I = 50.4 C = 54	n/a	I = 22(51 %), C = 22(47.8 %)	PEFR L-min-, FEV1 % pred, SF-121 physical component score, SF-121 mental component scoreCS, inhaled corticosteroids dosage, Systemic steroid dosage, Antileukotriene, exacerbations, unscheduled visits to hospital	In the intervention, mean SEM peak expiratory flow rate significantly increased at 4) and 6 months (compared to the control group. The intervention group also had better quality of life after 3 months, as determined using the Short Form-121 physical component score, and fewer episodes of exacerbation and unscheduled visits than the control group.
Rasumussen [37] (2013) Denmark	diagnosed on the basis of a combination of respiratory symptoms and at least one objective measurement of asthma (i.e., airway hyper responsiveness to inhaled methacholine of <4 mmol, peak expiratory flow [PEF] variability of >20 %, and/or a minimum of 15 % [300 mL] increase in FEV1 after bronchodilation)	I = 80 C = 85	I = 28 C = 30	N/A	I = 27(33.9 %) C = 30 (35.3.7 %)	Symptoms, AQLQ, FEV1 _300 mL, airway hyper responsiveness.	Improvement was found for the intervention group versus control for asthma symptoms Internet vs GP: odds ratio of 3.26; *P* < .001, AQLQ (odds ratio of 2.10, *P* = .04) lung function (odds ratio of 4.86, *P* < .001), airway hyper responsiveness. (odds ratio of 3.06, *P* = .02)

### Description of Interventions

A summary of the key components of the interventions is given in Table 2.

**Table 2:** Description of Interventions

Author (Year)	Mode of delivery	Health Education Included	Setting	Frequency of use	Theoretical basis included in paper	Duration
Van Gaalen [30]	Website/mobile phone	Yes	General practice	Daily	Yes (Chronic care model)	12 months
Van der Meer [31–33]						
Hashimoto [34]	Website	Yes	Hospital Outpatients	Daily	No	6 months
Bender [35]	Phone/Interactive Voice response	Yes	Community/clinic	2 or 3 calls in time period	Yes (Benefit risk model)	10 weeks
Liu [36]	Mobile Phone	Yes	Outpatient clinic	Daily	No	6 months
Rasmusen [37]	Website	Yes	General practice	Daily	No	6 months

#### Aim of intervention

Three of the interventions, led by Rasmussen and van der Meer (the SMASHING study) directly tested the impact of a digital intervention on asthma outcomes as a main objective [31–33, 36, 37]. Two studies focused on medication: Hashimoto et al. [34] assessed whether home monitoring of symptoms, lung function and fraction of exhaled nitric oxide facilitates tapering of oral corticosteroids and leads to reduction of corticosteroid consumption without worsening asthma control or asthma-related quality of life; Bender et al. [35] tested whether an interactive voice response system could improve adherence to controller medications.

#### Format and delivery

In the SMASHING study, participants accessed the intervention through a specially designed website, which allowed patients to report daily lung function (FEV_1_) values through the website or by text message [30–33]. In the Hashimoto et al. study, patients registered their daily FEV_1_ values, dose of oral corticosteroids and exhaled nitric oxide (FE_NO_) to an asthma monitoring service using an internet application or text messages [34]. Bender et al. used an interactive voice response system (IVR) through which participants received calls, and gave tailored responses based on information given by the participant [35]. Liu et al. used interactive self-care software installed on patients’ mobile telephones to record daily sleep quality, coughing severity, difficulties with breathing, activities affected by asthma, use of relievers, peak expiratory flow rate (PEFR) and PEFR variability [36]. Participants were loaned a mobile telephone if they did not have one or it was not compatible with the software. Rasmussen et al. used an internet-based asthma management tool; if the patient did not have access to a computer, a push-button telephone was used [37].

#### Education

All the studies provided additional education via the intervention. This was poorly described in some and very variable in content. Two studies described the education provided by the intervention: the SMASHING study [30–33] featured two group-based education sessions, which lasted 45 to 60 min, including exploration of a patient’s interests and previous knowledge (negotiating an agenda and patient-centred education), personalized feedback, and support for self-management (self-efficacy and implementing a plan for change); the Bender et al. study included information about a pre-existing free telephone service staffed by nurses capable of answering questions about asthma, and the Colorado Quit Line, offering free telephone based tobacco cessation information [35].

#### Additional health professional help available

Three of the studies specified that additional health professional help could be accessed through the intervention if participants required it. In the SMASHING study, intervention participants could communicate with a specialised asthma nurse if required [30–33]. In the Hashimoto et al. intervention a study nurse could monitor data entered at the web page and facilitate communication between patients and pulmonologists, if deemed necessary [34]. For the Rasmussen et al. intervention participants had access to a physician for treatment advice [37].

#### Setting

Two studies were conducted in general practice, 2 in outpatient clinics and one in a community clinic (see Table 2).

#### Duration and intensity

The SMASHING study was the longest intervention at twelve months [30–33]. The shortest was the Bender et al. study, which lasted 10 weeks [35]. The three remaining studies were all 6 months in duration. All studies required daily input by intervention participants with the exception of the Bender et al. study, in which participants received 2 or 3 telephone calls in the 10-week time period.

#### Theoretical basis for intervention included in paper

Two of the studies outlined a theoretical basis for their intervention. The SMASHING study used the Chronic Care model. This is aimed at improving health care outcomes for patients with a chronic disease by means of a proactive patient-professional partnership that addresses both organisational factors (such as decision support systems) and resources (such as self-management support) [30–33]. Bender et al. used the benefit risk model which posits that the probability of engaging in health-promoting behaviours depends on the person’s perception of risk and benefit related to the behaviour and its health consequence [35].

### Description of the study population

Characteristics of participants in the included studies are in Table 1, and showed considerable variation. Authors differed on how they defined asthma. The SMASHING study used physician-diagnosed asthma coded according to the International Classification of Primary Care in the electronic medical record and prescription of inhaled corticosteroids for at least 3 months in the previous year [30–33]. The sample in the Hashimoto et al. study had severe refractory asthma as defined by the American Thoracic Society [34]. Bender et al. used physician diagnosed asthma for which patients were prescribed daily inhaled corticosteroid treatment [35]. Liu et al. based their definition on clinical symptoms and physical examination, using the criteria for moderate to severe asthma as defined by the American Thoracic Society [36]. The Rasmussen et al. definition was based on a combination of respiratory symptoms and at least one objective measurement of asthma [37].

The percentage of male participants ranged from 28.9 % [30] to 49.4 % [36] and mean age ranged from 28 [37] years to 54.0 [36]. Only Bender et al. recorded the ethnicity of the participants [35]. Only van der Meer reported on socio-economic differences by recording the education status of participants [31].

### Quality appraisal

Details of the quality appraisal of the included studies can be found in Table 3. All the included studies were randomised controlled trials but two interventions were deemed to have an inadequate randomisation procedure [30, 31] and one was unclear on how the randomisation procedure took place [37]. Allocation concealment was only clear in two studies [35, 37]. One study had a dropout rate greater than 20 % [36]. Three of the studies did not control for any potential confounders in their analysis [35–37]. The majority of the studies were short in duration and relatively small in size, meaning that they were likely to be under-powered.

**Table 3:** Quality appraisal for included studies

Author (Year)	Appropriate Randomisation Technique	Allocation concealment	Dropout rate <20 %	Potential confounders properly accounted for	Were eligibility clear
Van Gaalen [30]	No	No	Yes	Yes	Yes
Van der Meer [31–33]					
Hashimoto [34]	No	No	Yes	Yes	Yes
Bender [35]	Yes	Yes	Yes	No	Yes
Liu [36]	Not clear	Not clear	No	No	Yes
Rasmusen [37]	Yes	Yes	Yes	No	Yes

## Outcomes

### Quality of life questionnaires

Four of the five studies reported on asthma specific quality of life indicators using the Juniper AQLQ questionnaire [30–35, 37] while Liu et al [36] used the Short Form (SF)-12 questionnaire to measure physical and mental health as general indicators of quality of life. Liu found that patients in the intervention group had a statistically significant improvement in physical health, with significantly higher physical health status than the control group at three months, and at the end of the intervention at six months. The mental health status (SF-12) of patients in the mobile phone intervention group did not significantly change throughout the study period. Patients in the control group showed a significant reduction in mental health status by the end of the study. Van der Meer et al. reported a significant improvement in the intervention group compared to usual care by the end of the intervention (difference 0.38 95%CI 0.20 to 0.56) [31]. Rasmussen et al. used odds ratios in measuring change in AQLQ and therefore was excluded from the meta-analysis. They found that those in the intervention group were twice as likely to show an improvement in AQLQ compared to the usual care group (OR 2.10, 95 % CI 1.02-4.31) [37].

**Fig. 2:**
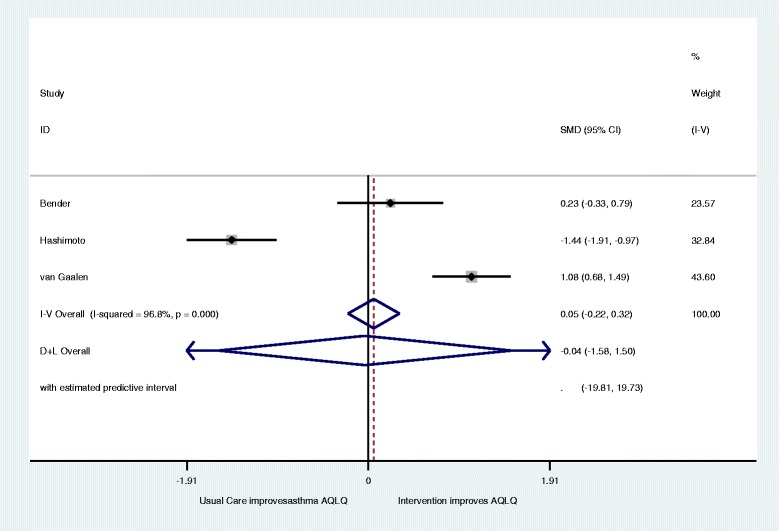
Forest plot of the effect of digital intervention on improvement in Asthma quality of life questionnaires (AQLQ)

**Fig. 3:**
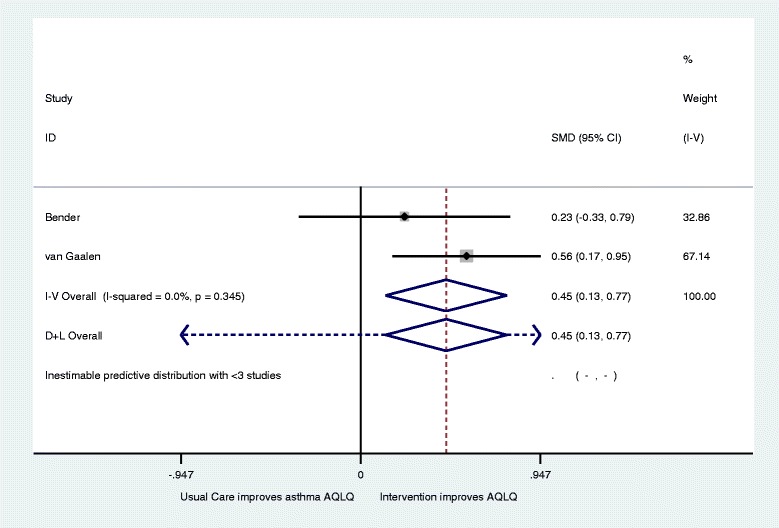
Revised forest plot of the effect of digital intervention on improvement in Asthma quality of life questionnaires (AQLQ)

This left three papers to be included in the meta-analysis with 123 intervention and 123 control patients (Fig. 2) [30, 34, 35]. This initial meta-analysis demonstrated no significant change in Asthma Quality of Life (AQLQ) (SMD 0.05; 95 % CI 0.32 to −0.22) with a high level of heterogeneity (I^2^ = 96.8; Q = *p* < 0.001). The Hashimoto et al. study^37^ was removed as its’ aim was to reduce the total dose of oral prednisolone without worsening control compared to the other two studies which was to improve asthma control. Figure 3 shows this reduced heterogeneity and the meta-analysis then showed significant improvement in AQLQ for intervention groups (SMD 0.45; 95 % CI 0.13 to 0.77: I^2^ = 0; Q = *p* = 0.34).

### Asthma control questionnaires

**Fig. 4:**
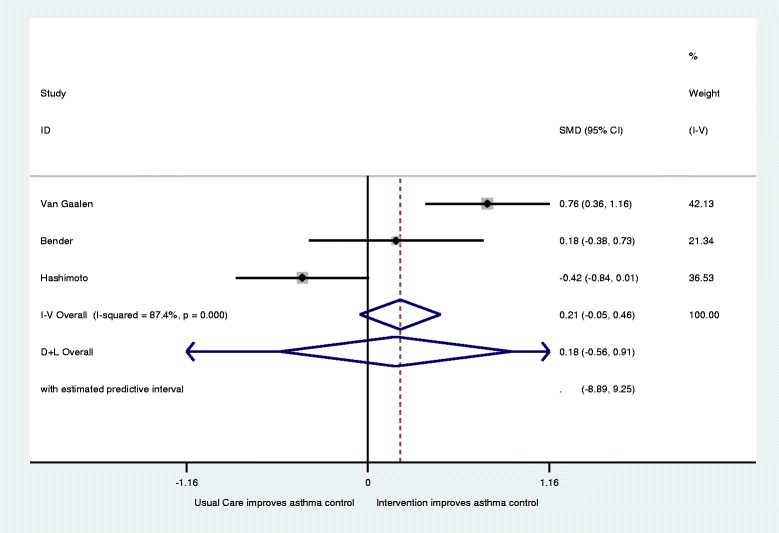
Forest plot for improvements in Asthma Control (increase indicates improvement)

**Fig. 5:**
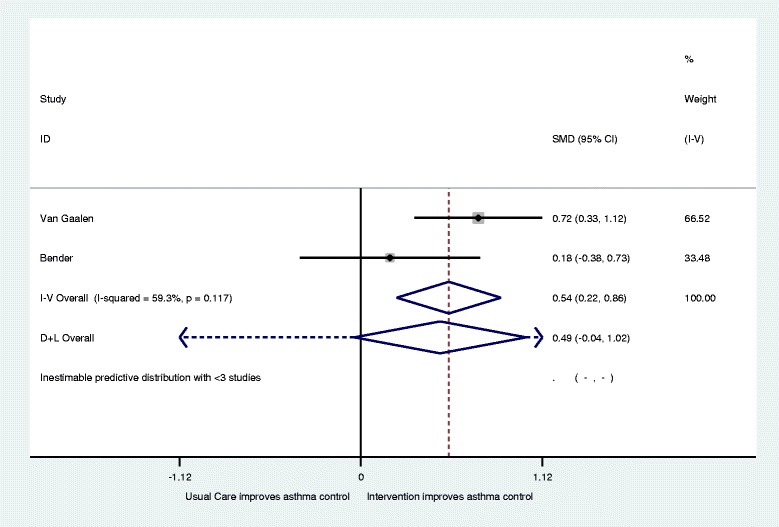
Revised forest plot for improvements in Asthma Control (increase indicates improvement)

Three of the interventions and five papers reported on measures of asthma control using a range of asthma control questionnaires (ACQ) [30–32, 34, 35]. Van der Meer et al. found for patients with well controlled asthma at baseline ACQ scores were not significantly different between the usual care and intervention group during follow-up. In patients with partly controlled asthma at baseline ACQ scores in the intervention group improved with −0.44 (95 % CI, −0.67 to −0.22) and −0.51 (− 0.73 to −0.29) after 3 and 12 months, respectively, compared to usual care. In patients with uncontrolled asthma at baseline ACQ scores in the intervention group improved with −0.57 (95 % CI, −0.84 to −0.31) and −0.82 (−1.10 to −0.55) after 3 and 12 months, respectively, compared to usual care and overall −0.47 (95 % CI, −0.64 to −0.30) after 12 months [31, 32]. This left three papers [30, 34, 35] included in a meta-analysis with 123 intervention and 123 control patients (Fig. 4). Overall they showed no significant difference in asthma control (SMD 0.21; 95 % CI −0.05 to 0 .46) with relatively high levels of heterogeneity recorded (I^2^ = 87.4; Q = *p* < 0.001). Again, after the removal of the Hashimoto et al. study [34], a small but significant improvement in ACQ was found for intervention patients with moderate heterogeneity recorded as shown by Fig. 5 (SMD 0.54; 95 % CI 0.22 to 0.86: I^2^ = 0.59.3;Q = *p* = 0.11).

### Forced expiratory volume in 1 second (FEV1)

Four studies examined changes in FEV1 [31, 34, 36, 37], although meta-analysis was not possible as some reported % predicted FEV1 [36] and others absolute FEV1 [31, 36, 37]. Van der Meer et al. found that mean prebronchodilator FEV1 changed by 0.24 L versus −0.01 L (adjusted difference, 0.25 [CI, 0.03 to 0.47 L] for the intervention and usual care groups, respectively [31]. Hashimoto et al. found no significant difference in changes in FEV1 between intervention and usual care groups [34]. Liu et al. used FEV1 % predicted and found that compared to the control group it significantly increased at 6 months (from 43.0 to 65.2 %predicted in the intervention group versus 46 to 56.5 %predicted in the control group *p* < 0.05) [36]. Rasmussen et al. found that those in the intervention group were significantly more likely to show an improvement in FEV1 of 300 mL or more at follow-up with 32 % in the intervention group improving compared to 9 % in the usual care group (OR 4.86; 1.97–11.94) [37].

### Other clinical outcomes

Liu et al. found that peak expiratory flow rate (PEFR) increased significantly from 4 months onwards in the intervention group, with the biggest difference recorded at the end of the six–month study (intervention group 382.7 L-min versus control group 343.5 L-min; *p* < 0.005) [36]. Rasmussen et al. found that 21 % of those in the intervention group compared to 8 % in the control group showed an improvement in airway responsiveness to inhaled methacholine by one or more dosage steps (OR 3.06 95 % CI 1.13–3.81) [37].

### Asthma symptoms

Van der Meer et al. found the number of asthma symptom free days showed a significant improvement in the intervention group from baseline to follow up (44.9 % to 63.1 %) compared to controls (44.5 % to 51.8 %) [31]. At follow up Rasmussen et al. found that 64 % of the intervention group showed an improvement in symptoms (as defined as improvement of one or more severity steps) compared to 35 % in the control group (OR 3.26 95 % CI 1.71–6.91) [37]. The severity of symptoms was graded as follows: very mild, respiratory symptoms less than once a week and nocturnal symptoms not more than twice a month; mild, respiratory symptoms 2 to 6 times a week and nocturnal symptoms more than twice a month but not weekly; moderate, respiratory symptoms daily and nocturnal symptoms more than once a week; and severe, respiratory symptoms constantly and nocturnal symptoms more than 4 times a week [37].

### Educational outcomes

Van der Meer et al. used the 12-item Consumer Asthma Knowledge Questionnaire [31] and found no significant differences in improvements in asthma knowledge or inhaler technique between intervention and controls [31] using the standardised checklist of the Dutch Asthma Foundation [38].

### Corticosteroids (inhaled or oral)

Four studies examined changes in use of corticosteroids, however due to using different measurements; a meta-analysis was not possible. Van Galen et al. found no significant difference in daily inhaled corticosteroid dose [30] while Van der Meer et al. also found that daily inhaled corticosteroid dose did not differ statistically significantly after 12 months [31]. Hashimoto et al. measured the cumulative reduction of oral corticosteroid exposure (actual cumulative dose minus the expected cumulative dose) [34]. The median cumulative sparing of prednisone equivalent after 6 months was significantly higher in the intervention group at 205 mg (25-75th percentile −221 to 777 mg) compared with 0 mg (−497 to 282 mg) in the control group (*p* = 0.02) [34]. In the Liu et al. study those in the intervention group significantly increased their mean daily dose of both systemic and inhaled corticosteroids from baseline, and no significant change was found in the control group. However, there was no significant difference between intervention and control groups at the end of the study period [36]. Rasmussen et al. found that significantly more patients in the intervention group used inhaled corticosteroids compared to the control group, increasing from 21 % at baseline to 91 % at follow up in the intervention group, compared with an increase from 17 % at baseline to 29 % at follow up in the control group [37].

### Other medications

Liu et al. reported that the percentage of patients treated with antileukotrienes was significantly higher in the intervention group at two months (intervention 60.5 % versus control 34.8 %; *p* = 0.015) but this difference had disappeared by the end of the study (intervention 39.5 % versus 34.8 %; *p* > 0.05) [36].

### Medication adherence

Van der Meer et al. found no significant difference between intervention and control groups in self-reported medication adherence [31]. Bender et al. found that mean inhaled corticosteroids adherence (determined by dividing the number of inhaler puffs taken each day by the number of puffs prescribed to be taken each day, and then averaged over the 10-week interval) was higher in the intervention than in the control group by a margin of 64.5 % to 49.1 % (*p* =0.03) [35]. The two groups also differed on the Belief in Medication Questionnaire, with the intervention group demonstrating a greater upward shift in positive medication beliefs, possibly explaining the improved adherence observed (*p* = 0.007) [35]. Rasmussen et al. found that compliance (defined as use of medication always or almost always) was significantly higher in the intervention group, with 87 % compliance compared with 54 % in the control group (*p* < 0.001) [37]. Rasmussen et al. also found that all intervention patients were on some form of asthma medication at follow up, compared to 74 % in the control group (*p* < 0.001) [37].

#### Exacerbations and health care utilisation

Van der Meer et al. found no significant differences in physician visits or telephone contacts with health provider between intervention and controls [31, 36]. Hashimoto et al. found no significant differences in exacerbations per patient or per year or days of hospitalisation between intervention and control groups [34]. Liu et al. reported fewer unscheduled visits and a lower number of patients visiting the emergency department in the intervention group versus the control group [36].

### Cost effectiveness

Van der Meer et al. reported that quality adjusted life years, as measured by the EuroQol-5D, did not significantly differ between the intervention and control group [32]. Costs of the Internet-based intervention were $254 per patient (95 % CI, $243 to $265) during the period of 1 year. Measuring from a societal perspective, the cost difference was $641 (95 % CI, $21957 to $3240), and from a health care perspective, the cost difference was $37 (95 % CI, $2874 to $950). At a willingness-to-pay of $50000 per QALY, the probability that Internet-based self-management was cost-effective compared to usual care was 62 % and 82 % from a societal and health care perspective, respectively [32].

## Discussion

This systematic review and meta-analysis of IDIs for self-management in adult asthma found only eight papers reporting five eligible studies. The studies were generally of moderate quality, small in size and short in duration, and used heterogeneous interventions. The quality and small number of included studies limits the conclusions from this review of IDIs. “The results were complicated further by the Hashimoto et al. study, whose aim was to investigate whether their intervention could facilitate tapering of oral corticosteroids in oral steroid dependent patients without worsening asthma control or asthma-related quality of life [34]. Inclusion of the Hashimoto study in the meta-analysis led to significant heterogeneity for both asthma control and asthma quality of life. Once the Hashimoto et al. study was removed, a small but significant improvement was found for both asthma control and AQLQ, albeit based on only two studies. Given the aim of the Hashimoto study it many be considered that the meta-analyses of the two remaining trials offers a more valid result. However, although the results of the revised meta-analysis were statistically significant it remained lower than 0.5 which is considered to be the minimal level for the difference to be clinically significant [38, 39] suggesting the impact of the interventions may be clinically limited. The effect of IDIs on other clinical outcomes is uncertain, due to the low number of studies and use of different metrics, which meant that meta-analysis was not possible. However, none of the studies reported significantly worse outcomes in the intervention groups for any indicator, with most reporting improvements in a range of other clinical outcomes.

This study builds on previous work [18] in looking at the impact of IDIs by using a more refined search strategy which focuses on adults only and using only interventions where the comparator group is usual care. This allows for the impact on adults with asthma to be shown more clearly then previous work which has included children [18]. The previous review included 19 unique RCTSs of which only two are included in this study [31, 37]. The small but significant improvement in for both asthma control and AQLQ, albeit based on only two studies, contrasts with evidence from other studies where no difference was found [18, 40]. In contrast to other reviews, two studies that analysed asthma symptoms showed statistically significant improvements for intervention patients [18, 40]. Results were mixed for changes in medication use, improvements in medication adherence, and health care utilisation. No improvement in asthma knowledge or reduction in exacerbations was reported. The small number of studies found is consistent with a recent systematic review of IDIs for asthma care which showed that only a small proportion of IDIs were aimed at adults with none including participants aged over 65 [18].

The review has a number of limitations. Only a small number of eligible studies were identified. The majority were of moderate quality, small in size and short in duration meaning that individual studies were likely to be under powered for most outcomes. The small number of studies also meant analysis of the possible effects of specific intervention components was not possible, limiting what the study can tell us about the effects of individual IDIs and how they might differ from each other. The studies were variable in length, with the longest lasting one year and the shortest just ten weeks. Short studies may underestimate the impact of the interventions since the performance of participants may continue to improve after the end of the intervention [41]. One follow up study included in this analysis showed that improvements in asthma-related quality of life and asthma control were sustained in participants for 1.5 years after the end of the intervention [30].

Included interventions were from a range of countries (Netherlands, USA, Taiwan and Denmark), suggesting that IDIs were suitable for use across a range of health systems. However, none of the trials were undertaken in low-income countries and there is no evidence on how intervention effects may differ by socio-economic status or ethnicity.

Only one study assessed the cost benefits of its impact, making the cost effectiveness of asthma IDI difficult to assess [32]. However, this study did suggest that the intervention could be supplied at similar costs to usual care, as well as offer additional benefits. Furthermore, the sustained improvements found in the follow up to this study suggest that the cost effectiveness may increase over a longer time period [32]. No studies examined how asthma IDIs may affect mental health.

## Conclusion

Digital self-management interventions for adults with asthma show potential for benefit, with evidence of improvements in some outcomes, and no evidence of harm. However, the evidence base remains weak, and it is not yet possible to recommend their use in clinical practice, due to the current lack of large, robust studies conducted and published.

## Consent for publication

Not applicable

## Availability of data and materials

Not applicable

## Ethics

No ethical approval was required.

## Additional file

Additional file 1: Asthma SR. Search strategy for Ovid MEDLINE(R) In-Process & Other Non-Indexed Citations and Ovid MEDLINE(R) 1946 to Present. (DOCX 17 kb)

## Abbreviations

ACQ: Asthma control questionnaires
AQLQ: Asthma Quality of Life
CI: Confidence Interval
FEV_1_: Forced expiratory volume in 1 second
IDIs: Interactive digital interventions
OR: Odds ratio
PEFR: Peak expiratory flow rate
RCTs: Randomised controlled trials
SF: Short form
SMD: Standardised mean difference
